# Effects of Temperature, Light and Digestive Fluid on the Stability of Major Arsenic Species in Antarctic Krill (*Euphausia superba*)

**DOI:** 10.3390/ani15213148

**Published:** 2025-10-30

**Authors:** Zhongquan Jiang, Haiyan Zhang, Yunyun Ji, Guangxin Yang, Cong Kong, Peng Wang, Tao Yuan, Xiaosheng Shen

**Affiliations:** 1 East China Sea Fisheries Research Institute, Chinese Academy of Fishery Sciences, Shanghai 200090, China; zhongquanj@sjtu.edu.cn (Z.J.);; 2 State Environmental Protection Key Laboratory of Environmental Health Impact Assessment of Emerging Contaminants, School of Environmental Science and Engineering, Shanghai Jiao Tong University, Shanghai 200240, China

**Keywords:** Antarctic krill (*Euphausia superba*), Arsenobetaine (AsB), in vitro digestion model, stability analysis

## Abstract

**Simple Summary:**

This study focuses on how storage conditions (temperature, light) and human digestion affect the stability of arsenobetaine (AsB), the main arsenic form in Antarctic krill (*Euphausia superba*). Krill samples stored for different durations underwent freezing, refrigeration, and room temperature treatments, with some additionally exposed to light. Results showed AsB stayed stable under refrigeration or short-term storage but dropped significantly after light exposure. In simulated digestion tests, gastric treatment reduced the more harmful arsenic form As(III) while boosting safe AsB levels. During intestinal digestion, two new arsenic forms emerged, though overall toxicity decreased. These findings support safe krill storage, processing, and related food safety guidelines.

**Abstract:**

Antarctic krill, an important marine resource, contains significant arsenic levels, predominantly as the low-toxicity arsenobetaine (AsB). However, the stability of AsB during post-harvest storage and its transformations during human digestion are poorly understood, which is critical for a comprehensive safety assessment. This research investigated the effects of temperature, light exposure, and in vitro simulated digestion on the stability and transformation dynamics of major arsenic species in Antarctic krill. The results revealed that AsB predominated among the arsenic species. AsB remained stable during long-term frozen storage (−18 °C for 5 months) and short-term refrigeration (4 °C for 2 days). However, AsB content decreased significantly during storage at ambient temperature (25 °C) and after prolonged light exposure (>8 h), indicating that improper storage conditions can lead to its degradation. During simulated gastrointestinal digestion, a significant transformation of arsenic species was observed. The content of toxic inorganic As(III) decreased significantly during the gastric phase, while the less-toxic AsB content markedly increased. Furthermore, dimethylarsinic acid (DMA) and As(V) were newly detected during the intestinal phase. These findings demonstrate that arsenic stability in krill is highly dependent on storage conditions. Moreover, the transformations during digestion—notably the decrease in As(III) and increase in AsB—suggest a potential reduction in overall arsenic toxicity upon consumption. This provides a critical theoretical basis for developing storage guidelines and improving human health risk assessments for Antarctic krill products.

## 1. Introduction

Antarctic krill (*Euphausia superba*), as a resource with enormous biomass and rich nutrition, had a catch of as much as 450,000 tons in the Southwest Atlantic in 2020, achieving the largest annual growth in three years [1]. Krill contains abundant crude protein (11.9–16.3%), crude fat (0.5–3.6%), and functional components like EPA/DHA and astaxanthin, making it the world’s largest polar biological protein reservoir [2,3]. Antarctic krill play a key role in the Southern Ocean ecosystem and are also an important target for fisheries. Therefore, in-depth research on their spatial distribution patterns and environmental influencing factors is the foundation for promoting the scientific management of this fishery [4,5]. In the 2023 and 2024 fishing season, the reported annual catch of Antarctic krill in the Southwest Atlantic increased to a record of approximately 500,000 tons [6]. However, environmental arsenic accumulates in Antarctic krill through both direct uptake and the food chain, jeopardizing product safety and posing major challenges to emerging industries [7]. This arsenic originates primarily from the natural environment and is enriched through the food chain, where inorganic arsenic in seawater is metabolized into organic forms like MMA and DMA, and ultimately into arsenobetaine (AsB) in zooplankton.

The total arsenic content in krill-derived products varies significantly: the detected value in krill powder is about 1.5 mg/kg, while krill oil, which is particularly noteworthy, can reach 6.5 mg/kg [8]. This finding has made krill oil an increasingly important subject of research in this field. Complementary research employing ICP-MS has further quantified total arsenic concentrations in Antarctic krill populations ranging from 4 to 14 mg/kg, collectively driving intensified toxicological research initiatives [9]. In summary, although the total arsenic content in Antarctic krill and its derived products exhibits seasonal and geographical variations, it remains at a relatively high level overall [10]. This poses a key constraint on the sustainable development of the Antarctic krill industry and complicates comprehensive safety assessments for its products.

A critical factor in this safety assessment is arsenic speciation, as arsenic toxicity is entirely dependent on its chemical form. Inorganic arsenic (e.g., As(III) and As(V)) is significantly more toxic than organic forms [11]. In marine organisms like Antarctic krill, the dominant form is typically arsenobetaine (AsB), a compound that exhibits very low acute toxicity [12,13,14]. However, this assumption hinges on a critical premise that AsB remains stable after harvest and during human consumption. It is unknown whether AsB can degrade or transform into more toxic inorganic species during post-harvest storage or during gastrointestinal digestion.

This concern is not merely theoretical. Studies show that in arsenic-affected areas, smoked fish had detectable arsenic levels below the national limit, unlike the uncontaminated control samples, suggesting the pollution likely occurred during processing [15]. Furthermore, research has demonstrated that external factors such as temperature, visible light, and ultrasonic irradiation exert a measurable influence on arsenic species transformation in aquatic environments [16,17]. Research has shown that different types of arsenic may transform into each other during the extraction process [18]. Although Antarctic krill contain high levels of total arsenic and exhibit diverse forms, studies on the bioavailability and stability of arsenic during storage, processing, and cooking are still scarce [19]. Crucially, the transformation dynamics of arsenic species during in vitro digestion, which determines the final arsenic profile entering systemic circulation, remains poorly characterized for krill products.

Therefore, this study aims to address this critical knowledge gap. We investigate the stability of major arsenic species during processing and in vitro digestion of Antarctic krill. We hypothesize that AsB stability is dependent on storage conditions, degrading significantly under ambient temperature and light exposure, and the acidic and enzymatic conditions of the stomach and intestine will induce significant transformations, altering the arsenic profile from what is ingested. Through controlled storage temperatures, light exposure, and simulated digestion, we investigated how external conditions affect arsenic species stability, establishing a theoretical basis for arsenic safety assessment and improving the risk–benefit analysis for the Antarctic krill resources.

## 2. Materials and Methods

### 2.1. Ethical Approval

All animal experiments were approved by the Laboratory Animal Welfare and Ethics Committee of Shanghai Jiao Tong University. The care and use of experimental animals were conducted in full compliance with Chinese animal welfare laws, guidelines, and policies (GB/T 35892-2018) [20].

### 2.2. Experimental Preparation

The Antarctic krill *(Euphausia superba)* originated from specimens captured in the Antarctic region. Immediately after being landed, they were frozen at −20 °C. Subsequently, upon arrival at the laboratory, they were stored at −80 °C. Prior to experimentation, samples were thawed at 4 °C for 4 h All krill samples under the same treatment group were combined and homogenized for 5 min using a homogenizer (T25 digital ULTRA-TURRAX, IKA, Staufen, Germany) under ice-water bath conditions (speed 12,000 rpm, with 30 s cooling intervals). The homogenization process maintained a consistent ratio of solid to liquid and extraction buffer. The obtained homogenate was divided into multiple parallel samples (*n* = 3) for subsequent analysis. The results of the total arsenic wet weight content determination of the divided samples showed a relative standard deviation (RSD) of less than 5%, demonstrating good uniformity of the homogenate, which was then stored at −18 °C until use. The Antarctic krill oil sample, supplied by Qingdao Kangjing Marine Biotechnology Co., Ltd. (Product Batch No. KO1903015, Qingdao, China), was stored at 4 °C upon arrival in the laboratory. The source of all Antarctic krill and oil samples is identical to that described above and will not be reiterated hereafter.

### 2.3. Storage Temperature Test

Triplicate 50 g aliquots of Antarctic krill samples were accurately weighed and stored under three conditions: (1) at −18 °C (frozen) for 0, 1, 2, 3, 4, and 5 months; (2) at 4 °C (refrigerated) for 0, 1, 2, 4, 6, and 8 days; and (3) at 25 °C (ambient temperature) for 0, 4, 8, 12, 18, and 24 h. Following storage, samples were evaluated for visual characteristics (color, odor, and texture) and subsequently analyzed for arsenic compound content and pH.

### 2.4. Light Exposure Experiment

Duplicate 50 g aliquots of Antarctic krill samples were accurately weighed and maintained at 4 °C in a thermostatic incubator (Model 164E from Thermo, Waltham, MA, USA). Samples were divided into two treatment groups: a light-exposed group under fluorescent illumination; a light-protected group stored in amber bottles with no light exposure. The lighting group uses Philips T8 type white daylight lamps (36 W/840, Signify, Amsterdam, The Netherlands) for illumination, with the light source positioned 30 cm from the surface of the sample, achieving an illuminance of 3500 ± 200 lux on the sample surface, in the wavelength range of 400 to 760 nm. Both groups were sampled at 0, 1, 2, 4, 8, 16, and 24 h. All lighting interval time points (a total of 7 time points) were independently subjected to 3 parallel experiments. At each interval, samples were assessed for visual attributes (color, odor, and physical state), followed by determination of arsenic compound content and pH.

### 2.5. In Vitro Simulated Digestion Experiment

The simulated gastrointestinal fluids were prepared in strict accordance with the methodology described by Thomas et al. [21]. For the gastric digestion experiment, exactly 0.50 g (precision ±0.01 g) of Antarctic krill oil was weighed into a 50 mL conical centrifuge tube, followed by the addition of 5 mL of simulated gastric fluid and pH adjustment to 2.0 ± 0.2. Samples were subjected to incubation in a thermostatic shaker (SG-8016A, Shuoguang, Beijing, China) at 37 °C and 100 r/min for 0, 1, and 2 h, respectively. The gastric juice digestion phase (time points 0, 1, and 2 h) included three parallels per group (*n* = 3). Following incubation, samples underwent pretreatment for arsenic speciation analysis prior to quantification of six arsenic compounds.

For the intestinal digestion experiment, exactly 0.50 g (precision ±0.01 g) of Antarctic krill oil was weighed into a 50 mL conical centrifuge tube. Following the addition of 5 mL simulated gastric fluid and digestion for a predetermined duration, 5 mL simulated intestinal fluid was introduced with subsequent pH adjustment to 8.0 ± 0.2. Samples were incubated in a thermostatic shaker at 37 °C and 100 r/min for 0, 2, 4, 6, and 8 h. The intestinal fluid digestion phase (time points 0, 2, 4, 6, 8 h) has 3 parallels in each group (*n* = 3). Following incubation, samples underwent pretreatment for arsenic speciation analysis (2.4.1.) and subsequent quantification of six arsenic compounds.

### 2.6. Sample Pretreatment

Briefly, 5.00 g (precision: ±0.01 g) of Antarctic krill sample was accurately weighed and placed into a 50 mL centrifuge tube; then, 20 mL of 1% HNO_3_ was added, and the mixture was homogenized. Following overnight immersion, extraction was performed in a 70 °C water bath for 2 h. After cooling to room temperature (25 ± 2 °C), 0.5 mL of 3% acetic acid was added and thoroughly mixed. Centrifugation (Hitachi 16RXII, Tokyo, Japan) was performed at 8000 r/min for 15 min, after storage at 4 °C for 5 min. Subsequently, 10 mL of supernatant was aliquoted and subjected to defatting extraction with 10 mL of n-hexane. Upon completion of centrifugation at 8000 r/min for 15 min, the upper phase was aspirated and discarded. This centrifugation step was repeated once. The solution was then filtered through a 0.22 μm membrane filter to finish the pretreatment workflow.

### 2.7. Methods for pH Determination

Accurately weigh out 5.00 g (precise to 0.01 g) of Antarctic krill sample into a 50 mL centrifuge tube. After storage under specified conditions, add 5 mL of ultrapure water (Merck Millipore, Burlington, MA, USA), vortex-mix thoroughly, and allow the mixture to stand for 30 min. Subsequently, collect the supernatant for pH determination.

### 2.8. Statistical Analysis

Experimental data were statistically analyzed using Microsoft Excel 2010 and SPSS 22.0 software. Significance analysis and multiple comparisons were conducted using one-way ANOVA followed by the Tukey HSD test. Descriptive statistics are presented as mean ± standard deviation ($\bar{x}\;$± SD), with statistical significance defined at α = 0.05.

## 3. Results

### 3.1. Predominant Arsenic Species in Antarctic Krill and Derived Products

**Figure 1:**
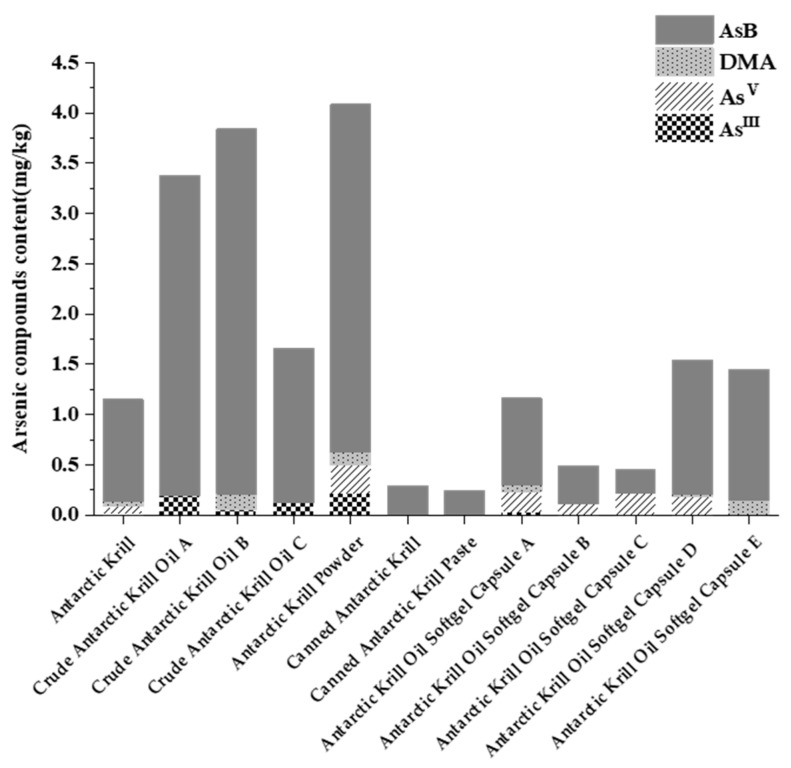
Distribution of different arsenic species wet weight content in Antarctic krill samples.

Arsenic speciation analysis was performed on twelve Antarctic krill and derived products, including frozen raw krill, krill meal, crude krill oil, and krill oil in capsules. As illustrated in Figure 1, while total arsenic content varied across samples, AsB was consistently identified as the predominant arsenic species, accounting for the vast majority (87.91–99.84%) of total arsenic in krill meal and canned krill products, and a slightly lower, though still dominant, proportion (51.94% to 94.50%) in krill oil products. In contrast, the more toxic inorganic arsenic (As(III) and As(V)) comprised only a minor fraction of the total. In the organic arsenic components, except for AsB and trace DMA, the content of MMA and AsC in all samples was below the quantification limit of this method (LOQ = 0.03 mg/kg) and was not detected. It is important to note that while MMA and AsC were ‘not detected’, this is relative to the method’s LOQ. Their presence at trace levels below 0.03 mg/kg, or the presence of other unquantified arsenosugars, cannot be ruled out and may be relevant to the transformations observed later during digestion.

### 3.2. Effect of Storage Temperature on AsB Stability in Antarctic Krill

**Figure 2:**
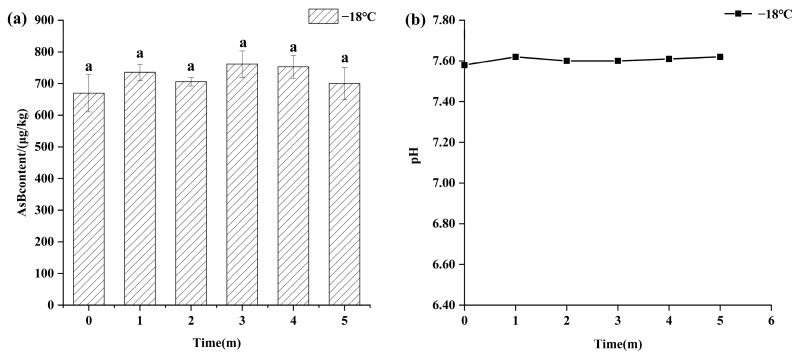
Changes in AsB content (**a**) and pH (**b**) of Antarctic krill during frozen storage (−18 °C) (*n* = 3). Identical lower letters indicate no significant (*p* < 0.05) difference.

Temperature was a critical factor in AsB stability. As shown in Figure 2, samples stored under frozen conditions (−18 °C) for 5 months exhibited high stability, with no statistically significant changes (*p* > 0.05) in either AsB content (655.76 ± 59.15 μg/kg) or pH (7.60 ± 0.03).

**Figure 3:**
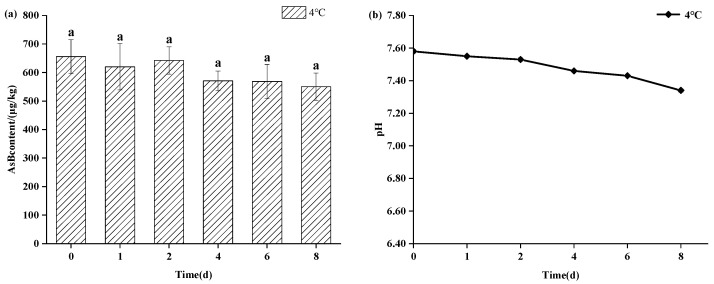
Changes in AsB content (**a**) and pH (**b**) of Antarctic krill during cold storage (4 °C) (*n* = 3). Identical lower letters indicate no significant (*p* < 0.05) difference.

**Figure 4:**
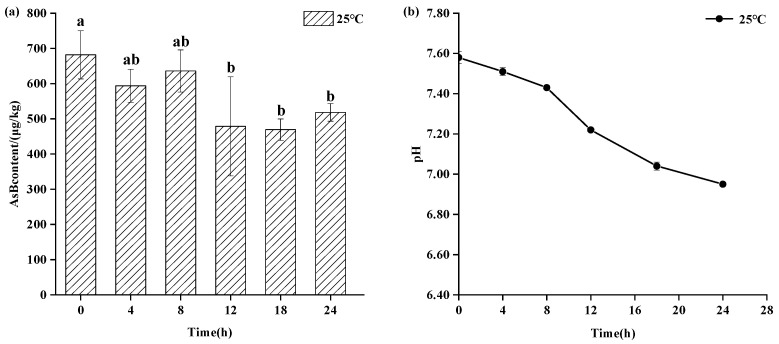
Changes in AsB content (**a**) and pH (**b**) of Antarctic krill during room storage (25 °C) (*n* = 3). Different lower letters indicate significant differences (*p* < 0.05) in AsB content among Antarctic krill stored at room temperature for varying durations.

As shown in Figure 3, refrigerated storage (4 °C) also maintained AsB stability for up to 8 days, showing no statistically significant change (*p* > 0.05) in its content. However, a gradual decline in pH was observed at this temperature, suggesting the onset of sample deterioration even as AsB remained chemically stable. As shown in Figure 4, in stark contrast, storage at ambient temperature (25 °C) induced rapid degradation. While AsB content was stable for the first 4 h, a significant decrease (*p* < 0.05) was observed after 12 h.

As shown in Figure 4, Antarctic krill samples stored at ambient temperature (25 °C) for 24 h exhibited changes in Arsenobetaine (AsB) content and pH. While a change in AsB content was observed after 4 h, it was not statistically significant (*p* > 0.05). However, a significant decrease in AsB content occurred after 12 h (*p* < 0.05). Concurrently, the pH decreased from 7.58 ± 0.02 to 6.95 ± 0.01.

### 3.3. Effect of Illumination on AsB Stability in Antarctic Krill

**Figure 5:**
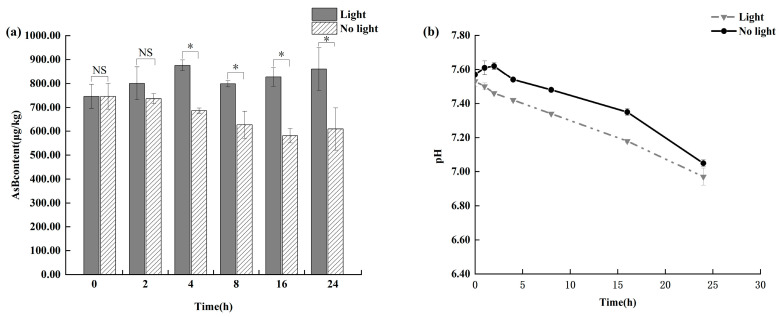
Changes in AsB content (**a**) and pH (**b**) in Antarctic krill under light and no light (*n* = 3). “*” and “NS” determine whether there is a significant difference in AsB content between the light-exposed group and the non-light group under the same time condition. (* *p* < 0.05; NS, not significant.).

As shown in Figure 5, light exposure showed a clear time-dependent effect on AsB degradation. Initially, no statistically significant changes (*p* > 0.05) were observed in AsB content in either the light-exposed or light-shielded group within the first 8 h of storage. However, a significant degradation pattern emerged beyond 8 h. As Antarctic krill quality deteriorated, the light-exposed group exhibited a statistically significant decrease in AsB content (*p* < 0.05). In contrast, the light-shielded group showed significantly greater stability, maintaining levels comparable to those observed under refrigeration.

### 3.4. Effect of In Vitro Digestion Simulation on the Stability of Major Arsenic Species in Antarctic Krill Oil

**Figure 6:**
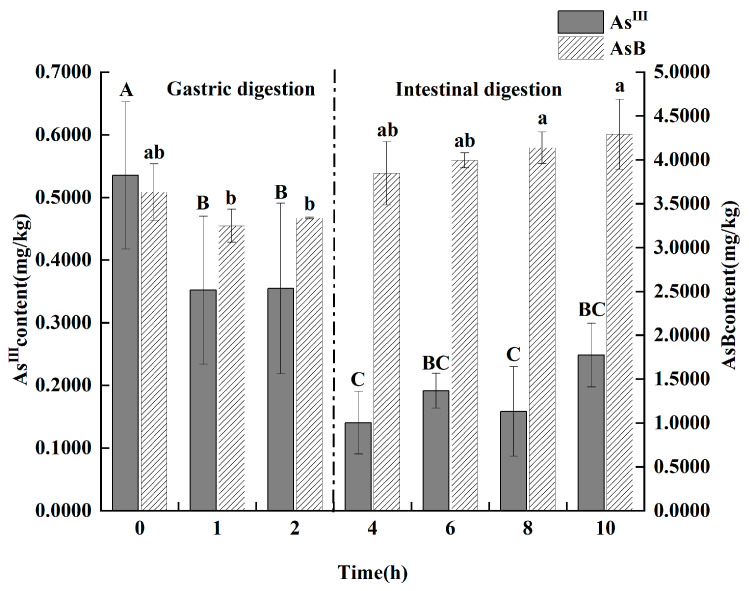
Effect of gastrointestinal fluid digestion time on the contents of As(III) and AsB in Antarctic krill oil (*n* = 3). Different capital and lower letters indicate significant (*p* < 0.05) differences in the content of As(III) and AsB at different digestion times. The data in the charts of the manuscript are mean ± standard deviation (SD).

As shown in Figure 6, the simulated gastrointestinal digestion induced significant and rapid transformations of arsenic species. During the gastric phase, two key changes occurred simultaneously: the content of toxic As(III) exhibited a significant decrease from 535.25 ± 117.67 μg/kg to 354.1 ± 58.18 μg/kg (*p* < 0.05), while the low-toxicity AsB content demonstrated a highly significant increase (*p* < 0.01). Upon entering the intestinal phase, this transformation continued. As(III) levels remained suppressed. Crucially, after 2 h of in vitro intestinal digestion, two arsenic species that were not present in the original oil, DMA and arsenate [As(V)], were detected at concentrations of 260.35 ± 67.35 μg/kg and 14.75 ± 1.55 μg/kg, respectively.

## 4. Discussion

### 4.1. Compliance and Speciation of Arsenic in Antarctic Krill Products

Compliance with arsenic limits in Antarctic krill and its products is contingent upon both the speciation and concentration of arsenic. According to the current National Food Safety Standard of China (GB2762-2022) [22], only inorganic arsenic in krill oil and its products is regulated (≤0.5 mg/kg), with no specification for total arsenic content. Institutions such as the EU and CAC have not set limits for total arsenic/inorganic arsenic in aquatic products. Australia has a limit of 2.0 mg/kg for inorganic arsenic in fish and shellfish, while the United States has limits of 76 mg/kg and 86 mg/kg for total arsenic in shellfish and mollusks, respectively. Compared to the standards in Australia and the United States, China’s limits on arsenic in aquatic products are stricter. However, according to WHO standards, the maximum allowable intake of arsenic for adults is 2.1 µg/kg of body weight per day. The arsenic ADMI in the human body is below the limit value of arsenic. Consequently, all 12 Antarctic krill products analyzed in this study contained inorganic arsenic below 0.5 mg/kg, meeting regulatory requirements. Critically, this study confirms AsB as the predominant arsenic species in these products. This conclusion demonstrates high reliability, as it is corroborated by previous findings obtained using HPLC/ICPMS methodologies [23]. The trivalent metabolites (MMA and DMA) produced by the metabolism of arsenic in the body can adversely affect multiple organ systems, including the immune system, and their toxic effects are similar to those of inorganic arsenic [24]. Therefore, regulating solely inorganic arsenic content is insufficient; controlling total arsenic levels is also warranted. Furthermore, a comprehensive safety evaluation of arsenic in Antarctic krill necessitates research into the stability of major arsenic species during storage, processing, and trophic transfer within the food chain.

### 4.2. Influence of External Environments on Arsenic Speciation in Antarctic Krill

The stability and transformation of arsenic species, particularly AsB, in Antarctic krill and its products are critically influenced by external environmental factors encountered during storage, processing, and consumption. Understanding the interplay between these factors—notably temperature fluctuations, light exposure, and the biochemical conditions of digestion—is essential for assessing product quality, arsenic speciation dynamics, and potential toxicological risks. This section synthesizes experimental findings on the impact of these key external drivers.

Firstly, Antarctic krill undergoes rapid decomposition of muscle proteins after death due to its extremely active autolytic enzyme system, which is accompanied by the production of peptides and free amino acids [25,26]. Antarctic krill undergoes immediate activation of protein hydrolases upon death, triggering rapid tissue autolysis that accelerates quality deterioration. The amino acids liberated by proteolytic degradation of muscle tissue at this stage influence the pH of shrimp meat. An increase in the liberation of basic amino acids elevates the pH, while an increase in acidic amino acids lowers it. Therefore, pH value can serve as a potential indicator for assessing the freshness of Antarctic krill. Data indicate that krill samples stored at −20 °C exhibited a significantly higher pH value (7.77) compared to those stored at −40 °C (7.47) and −80 °C (7.40) [27].

Temperature emerged as a primary determinant of both quality parameters and arsenic speciation stability. Frozen storage (−18 °C) effectively preserved sensory attributes (pink color, firm texture, absence of off-odors), which remained largely consistent with those observed prior to freezing. Therefore, frozen storage not only preserves its quality but also maintains the stability of arsenic speciation. Concurrently, Antarctic krill samples stored under refrigeration (4 °C) for 8 days exhibited slight browning, mild off-odors, softening of texture, and diminished elasticity. Experimental results demonstrated that Antarctic krill samples maintained acceptable quality within the initial 2 days of refrigerated storage, with minimal pH variation. Although a reduction in AsB content was observed, it was not statistically significant. Beyond 2 days of refrigeration, samples exhibited increasingly pronounced sensory deterioration accompanied by a progressive pH decline and marked quality degradation. This deterioration is likely attributable to proteolytic degradation of muscle tissue, liberating elevated levels of acidic amino acids.

Compared to the two aforementioned storage temperatures, Antarctic krill stored at ambient temperature (25 °C) exhibited pronounced browning, significant tissue softening, development of off-odors (notably fishy and ammonia notes), and more rapid quality deterioration. The degradation of muscle glycogen generates lactic acid, inducing a pronounced pH shift within a relatively short timeframe. Instead of merely observing a reduction, we interpret this as a mechanistic link: the significant reduction (*p* < 0.05) in AsB content observed after 12 h occurred concurrently with this rapid quality degradation and pronounced pH shift. This suggests that the mechanism for AsB degradation at ambient temperature is not simple thermal decay, but rather a complex process facilitated by the acidic and enzyme-rich environment created by rapid autolysis.

Light exposure effects on AsB speciation exhibited a distinct time dependence. Experimental results demonstrate that short-term light exposure exerts no significant effect on AsB speciation transformation, whereas prolonged illumination (>8 h) significantly influences AsB conversion. This indicates that light exposure facilitates AsB transformation, potentially through light-induced thermal effects, accelerating Antarctic krill quality deterioration and subsequent pH shifts, collectively promoting AsB conversion. Previous research on oxidative removal of As(III) from groundwater demonstrated that light conditions promote As(III) oxidation to As(V) [28]. These findings collectively indicate that illumination facilitates not only inorganic arsenic speciation transformation but also exerts a measurable influence on the transformation of organic arsenic species.

The ultimate fate of ingested arsenic species, however, is determined within the digestive system. In vitro digestion experiment strongly suggests that following gastrointestinal digestion, As(III) undergoes methylation to form DMA, with partial decomposition yielding minor quantities of As(V). These findings align with previous studies investigating similar arsenic transformations [29].

A key finding of the in vitro digestion was the significant increase in AsB content during the gastric phase, a phenomenon that requires interpretation as it suggests a transformation or release mechanism. We propose two primary speculative mechanisms for this observation. First, the AsB in the original krill oil may not be fully bioaccessible for extraction; it could be bound within the complex lipid and protein matrix. The harsh, acidic, and enzymatic conditions of the gastric fluid may break down this matrix, liberating the bound AsB and thus increasing its detectable concentration in the digestate. Second, it is possible that other unquantified organoarsenic precursors, such as arsenosugars, are chemically converted into the more stable AsB end-product under gastric conditions. In either case, this finding highlights that the AsB concentration measured in the raw product may not fully represent the amount released during digestion.

In seafood, the typical order of arsenic compound toxicity is: As(III) > As(V) ≫ DMA, MMA ≫ arsenosugars, AsC, AsB [30]. After in vitro gastrointestinal digestion of Antarctic krill oil samples, the observed transformations—the decrease in highly toxic As(III), the conversion into the less toxic DMA, and the marked increase in low-toxicity AsB—collectively suggest that the digested Antarctic krill oil exhibited a trend towards reduced overall toxicity. Consequently, the digested Antarctic krill oil exhibited a trend towards reduced overall toxicity. This suggests a potential decrease in health risks associated with human consumption. However, the exact toxicological implications require further comprehensive investigation.

### 4.3. Future Perspectives

The in vitro simulated gastrointestinal digestion model used in this study has limitations when extrapolating the results to real human conditions. Firstly, the model lacks an absorption process. In vivo, arsenic species are continuously absorbed by the intestinal epithelium, altering their gastrointestinal concentrations and affecting transformation equilibria. Consequently, the amounts detected at the end of digestion may not fully represent actual absorption levels. Secondly, the model utilized standardized enzyme and pH protocols, failing to fully replicate the complex physiological environment of the human gut, such as variations in bile salts, the role of the mucus layer, and intricate gut microbiota interactions. Furthermore, the omission of distinct microbial communities means not all potential transformation pathways were captured. Therefore, the observed decrease in As(III) and increase in DMA and AsB should be interpreted as indicative of transformation trends rather than definitive quantitative results. Future research should employ in vivo models or advanced co-culture systems incorporating intestinal cells and microorganisms to verify these findings and provide a more comprehensive basis for human health risk assessment.

From a food safety perspective, although AsB exhibits relatively low toxicity, it may degrade or convert into more toxic inorganic arsenic species, such as As(III), under conditions like long-term storage, light exposure, or cooking processes, thereby posing a potential threat. The observed transformation pathways, including the methylation of As(III) to DMA and the increase in AsB, indicate that while AsB itself is less toxic, the bioavailability, absorption rates, and chronic health effects of the newly formed arsenic species remain unknown. Thus, it is crucial to conduct in vivo studies tracking the absorption, distribution, metabolism, and excretion (ADME) of these transformed arsenic species to adequately characterize the risks associated with consuming Antarctic krill products.

## 5. Conclusions

This study utilized HPLC-(UV)-HG-AFS to analyze the arsenic species in Antarctic krill, finding arsenobetaine (AsB) to be the dominant form (86.51–99.84%). It was observed that AsB remained stable under short-term storage conditions (−18 °C for 5 months, 4 °C for 2 days, and 25 °C for 2 h). However, extended storage at ambient temperature (25 °C) and light exposure led to significant AsB reduction. This highlights the critical importance of maintaining a strict cold chain and protecting krill products from light to prevent the degradation of AsB. During simulated gastrointestinal digestion, As(III) levels decreased while AsB increased, indicating a transformation of arsenic species that could potentially reduce toxicity. This finding has direct implications for public health, suggesting that risk assessments based solely on the inorganic arsenic content of the raw product may be overly conservative, as the digestive process itself appears to mitigate toxicity. Our findings provide valuable insights for the safe utilization and risk assessment of Antarctic krill resources.

## Data Availability

Data are contained within the article.
